# Anti-Leishmanial and Cytotoxic Activities of a Series of Maleimides: Synthesis, Biological Evaluation and Structure-Activity Relationship

**DOI:** 10.3390/molecules23112878

**Published:** 2018-11-05

**Authors:** Yongxian Fan, Yuele Lu, Xiaolong Chen, Babu Tekwani, Xing-Cong Li, Yinchu Shen

**Affiliations:** 1 Institute of Fermentation Engineering, College of Biotechnology and Bioengineering, Zhejiang University of Technology, 18# Chaowang Road, Hangzhou 310032, China; lilyfan@zjut.edu.cn (Y.F.); luyuele@zjut.edu.cn (Y.L.); syc@zjut.edu.cn (Y.S.); 2 National Center for Natural Products Research, Research Institute of Pharmaceutical Sciences and Department of BioMolecular Sciences, School of Pharmacy, The University of Mississippi, University, MS 38677, USA; btekwani@olemiss.edu (B.T.); xcli7@olemiss.edu (X.-C.L.)

**Keywords:** anti-leishmanial activity, *Leishmania donovani*, maleimides, cytotoxicity, SAR

## Abstract

In the present study, 45 maleimides have been synthesized and evaluated for anti-leishmanial activities against *L. donovani* in vitro and cytotoxicity toward THP1 cells. All compounds exhibited obvious anti-leishmanial activities. Among the tested compounds, there were 10 maleimides with superior anti-leishmanial activities to standard drug amphotericin B, and 32 maleimides with superior anti-leishmanial activities to standard drug pentamidine, especially compounds **16** (IC_50_ < 0.0128 μg/mL) and **42** (IC_50_ < 0.0128 μg/mL), which showed extraordinary efficacy in an in vitro test and low cytotoxicities (CC_50_ > 10 μg/mL). The anti-leishmanial activities of **16** and **42** were 10 times better than that of amphotericin B. The structure and activity relationship (SAR) studies revealed that 3,4-non-substituted maleimides displayed the strongest anti-leishmanial activities compared to those for 3-methyl-maleimides and 3,4-dichloro-maleimides. 3,4-dichloro-maleimides were the least cytotoxic compared to 3-methyl-maleimides and 3,4-non-substituted maleimides. The results show that several of the reported compounds are promising leads for potential anti-leishmanial drug development.

## 1. Introduction

Leishmaniasis, which is caused by several species of *Leishmania*, is one of the major tropical diseases defined by the World Health Organization (WHO), affecting about 12 million people [1,2]. A wide range of clinical manifestations are encompassed, including visceral leishmaniasis, cutaneous leishmaniasis, and mucocutaneous leishmaniasis. Among them, visceral leishmaniasis is the most severe form of the disease. Visceral leishmaniasis, also called black fever or Kala-azar, has a fatality rate as high as 100% within two years if untreated, and spontaneous cure is extremely rare [3,4]. Visceral leishmaniasis is found throughout the intertropical and temperate regions, and threatens around 350 million people in 88 countries. Up to now, few medicines have been available for leishmaniasis. Pentostam is the most widely used drug, which contains the heavy metal antimony. Other medicines, such as amphotericin B and its derivatives [5], liposomal amphotericin B [5], paromomycin [6], and miltefosine [7], have their individual problems, such as toxicity, poor efficacy, or high cost. Meanwhile, the emerging drug-resistant parasites have caused further problems for therapy of the disease. Therefore, the discovery of new types of medicines with novel chemical structures is highly desirable. 

Recently, other candidate compounds, including buparvaquone [8], aminoquinolines [9], peptoids [10], (4-arylpiperazin-1-yl)(1-(thiophen-2-yl)-9H-pyrido[3,4-b]indol-3-yl) methanone derivatives [11], amino acid-triazole hybrids [12], biscoumarins [13], triazolyl quinoline derivatives [14], benzopiperidine, benzopyridine and phenyl piperazine based compounds [15], 2,5-diarylidene cyclohexanones [16], natamycin [17], piperazinyl-β-carboline derivatives [18], thiosemicarbazones [19], and so on, were evaluated and investigated. But for most of them, the IC_50_s of anti-leishmanial activities still remained to be in micromolar ranges except thiosemicarbazones (two compounds had the IC_50_s of 0.060 μg/mL and 0.068 μg/mL against promastigotes of *L. major*.) [19].

Maleimides, including natural products with maleimide core moiety, had excellent biological activities, including antimicrobial [20,21,22,23,24,25,26,27,28] and enzyme inhibition activities [29,30,31]. *N*-(4-Fluorophenyl)-dichloro-maleimide, significantly inhibiting fungal growth, was first developed and used to control the diseases of apple scab, rice blast and tomato late blight [32]. The MICs of *N*-butyl-maleimide and *N*-(4-phenylbutyl)-maleimide against 10 fungi were in the range of 0.48–15.63 μg/mL, similar to that of ampicillin, and had little toxicity to humans [33]. The antimicrobial mechanism was investigated and found that maleimides could interact preferably with the hydrophobic domains of target enzymes resulting in inactivation of sulfhydryl groups [29], which were essential for their catalytic activities. The activities were greatly affected by the structure of C=C double bond in the circle of diimide. For instance, *N*-ethyl-maleimide (NEM) and *N*-*tert*-butyl-maleimide inhibited β-(1,3)-glucan synthase with IC_50_ values 8.5 ± 1.1 μg/mL, 13.7 ± 2.3 μg/mL, respectively, and then influenced microbial growth [34]. In addition to great antimicrobial activities, maleimides had also been widely researched in medicine as antianxiety [35], anti-inflammatory [36], anticancer [37,38] and neuroprotective agents.

However, it should be noted that the reported studies were mainly focused on antimicrobial activity. No anti-leishmanial activity of maleimides has been reported before this. In the present work, a series of *N*-substituted maleimides, methyl-maleimides, and dichloro-maleimides were synthesized, and their anti-leishmanial effects in vitro and cytotoxicity toward THP1 cells were investigated. The results suggest that some of the synthesized maleimides might be developed as the anti-leishmanial drugs in the future.

## 2. Results and Discussion

### 2.1. Chemistry

**Scheme 1:**
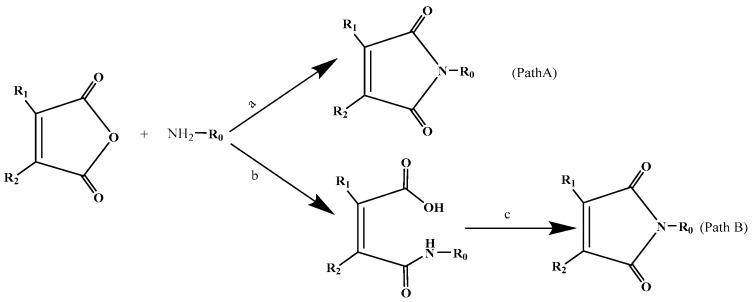
Synthesis of *N*-substituted maleimide derivatives. Path A: a. CH_3_COOH. Path B: b. toluene, 25–65 °C, 2–8 h; c. CH_3_COONa, (Et)_3_N, 101 °C, 10–24 h.

All maleimides were synthesized employing two methods according to an improved procedure based on the reported methods [22,32,33], using amines and maleic anhydrides as starting materials (Scheme 1). Path A was a facile method by one-step reaction to prepare 3,4-dichloromaleimides and 3-methylmaleimides with shorter reaction time, especially for synthesis of the former. However, path B was used to prepare *N*-alkylmaleimides (**1**–**4**) by two-step reactions, dehydration and ring-closing reaction. Furthermore, path A had higher yields and an easier isolation method when compared to path B. The desired compound could be easily synthesized in good yield (more than 70%) in one or two steps, as either crystalline or oily compound.

### 2.2. Biological Evaluations

#### 2.2.1. Anti-leishmanial Activity

**Figure 1:**
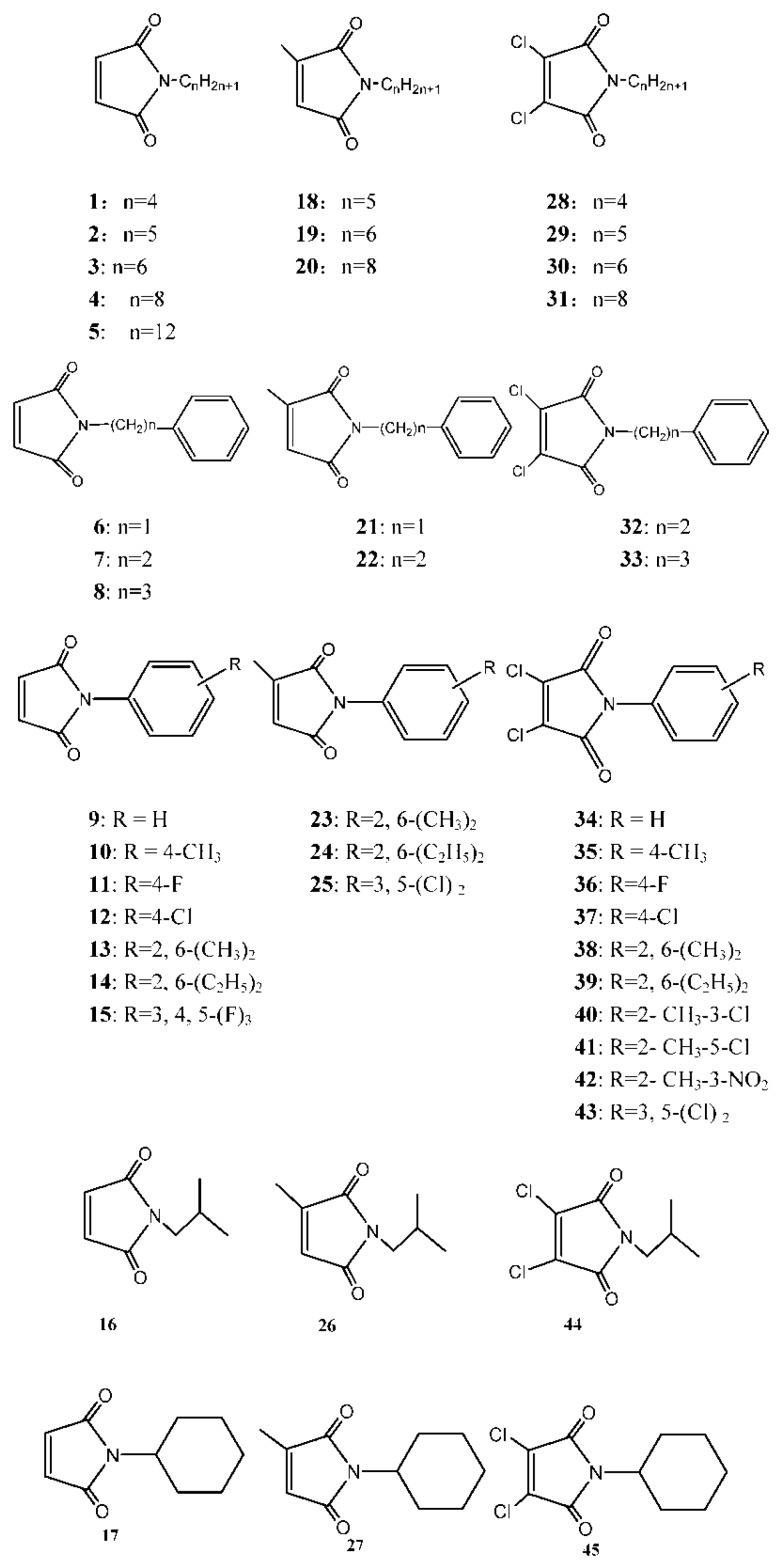
Chemical structures of the studied *N*-substituted maleimides.

**Figure 2:**
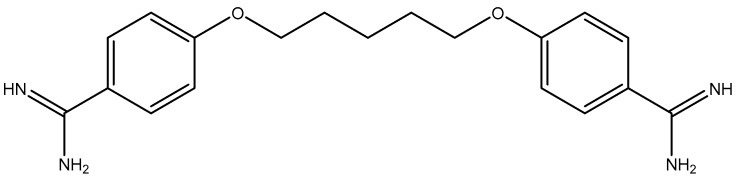
The chemical structure of pentamidine.

**Figure 3:**
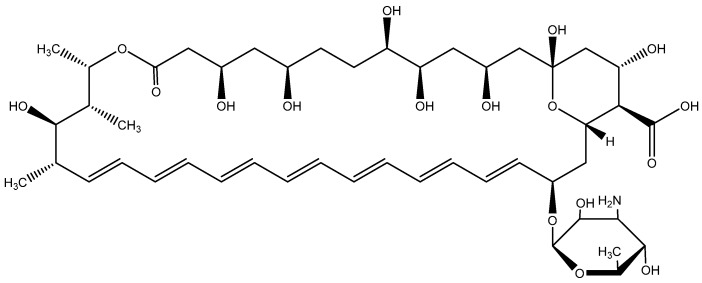
The chemical structure of amphotericin B.

45 maleimides [20,29,32,33] (Figure 1) and two drugs, pentamidine (Figure 2) and amphotericin B (Figure 3), were evaluated against *L. donovani*, to identify the most active compounds that are worthy of further investigation (Table 1). Anti-leishmanial activity in vitro was described in terms of IC_50_, which is the effective concentration (μg/mL) required to achieve 50% growth inhibition, with promastigotes in their exponential growth phase. Most of tested compounds had good anti-leishmanial activities (IC_50_s for 41 compounds were less than 1 μg/mL). Among them, the IC_50_s of **16** and **42** were less than 0.0128 μg/mL, much less than those of amphotericin B (IC_50_ = 0.12 μg/mL) and pentamidine (IC_50_ = 0.64 μg/mL), which reached nanogram grade. These activities were stronger than most compounds in literature [8,9,10,11,12,13,14,15,16,17,18,19]. There were another eight candidate compounds (**1**, **3**, **6, 7**, **8**, **13**, **14**, and **41**) which had better activities than amphotericin B.

#### 2.2.2. Cytotoxicity

In order to verify the safety of maleimides, they were tested for cytotoxicity against human monocytic leukemia cells (THP1) 50% cytotoxic concentration values represent the concentration of compound required to kill 50% of the THP1 cells were calculated (CC_50_). The selectivity indices were calculated using the formula SI = CC_50_ /IC_50_, against promastigotes. Interestingly, CC_50_s of 31 out of 45 maleimides were greater than 10 μg/mL, which highlighted their safety on mammalian cells (Table 1). SIs of **16** and **42** were greater than 781.3.

### 2.3. Structure-Activity Relationships

#### 2.3.1. Influences of Substituents at the 3- and 4-Positions of the Maleimide Ring

As shown in Table 1, the introduction of substituents at the 3- and 4-positions on the maleimide ring had different influences on the anti-leishmanial activities against *L. donovani*, depending on the type of introduced substituents. In general, 3,4-non-substituted maleimides (**1**–**17**) displayed very strong anti-leishmanial activities, with IC_50_ values ranging from 0.0128 to 2.21 μg/mL, 0.336 on the average, comparing to 1.774 μg/mL for 3-methyl-maleimides and 0.501 μg/mL for 3,4-dichloro-maleimides. Especially, **1**, **3**, **6, 7**, **8**, **13**, **14**, and **16** showed more interesting anti-leishmanial activities than the corresponding 3-methyl-maleimides and 3,4-dichloro-maleimides, which were superior or much superior to amphotericin B (IC_50_ = 0.12 μg/mL). In them, 15 compounds had better activities than pentamidine (IC_50_ = 0.64 μg/mL) except **5** (IC_50_ = 2.21 μg/mL) and **12** (IC_50_ = 1.02 μg/mL). Moreover, **16** and **42** displayed the strongest anti-leishmanial activity (IC_50_ < 0.0128 μg/mL). However, there was no apparent regularity for the influences of variation in substituents at the 3- and 4-positions on the maleimide ring. As to cytotoxicity, 3,4-dichloro-maleimides were least cytotoxic, whose CC_50_s were all higher than 10 μg/mL. Furthermore, 3-methyl-maleimides (**18**–**27**) were the most cytotoxic, whose CC_50_s were all less than 10 μg/mL.

#### 2.3.2. Influences of the *N*-Substituents

*Influences of variation in the alkyl chain length*. Results in Table 1 show that the *N*-alkyl substituents (**1** to **5**, **18** to **20** and **28** to **31**) had influences on anti-leishmanial activity. As the length of the *N*-alkyl chain increased, the anti-leishmanial activity decreased significantly. However, **1** exhibited the highest anti-leishmanial activity, with an IC_50_ of 0.08 μg/mL, which was one of the compounds with the best activity. It could possibly be explained that with the change in the polarity and *N*-alkyl chain length, their ability to connect enzymes differs. Therefore, with variation in chain length, **1** to **5**, **18** to **20** and **28** to **31** had different influences on anti-leishmanial activities. It was observed that *N*-phenylalkyl substituents (**6** to **8**, **21** to **22** and **32** and **33**) showed a correlation with the anti-leishmanial activity against *L. donovani*. As the alkyl chain length of *N*-phenylalkyl substituents increased, the anti-leishmanial activities decreased gradually (except for **33**), which might be explained by the N-C distance between the two rings playing an important role in the anti-leishmanial activities of these compounds. 

*Influences of the substituted benzene ring.* As for compounds with a mono-substituent on position 4 of phenyl ring, such as **10** to **12**, and **35** to **37**, it was obvious that the different groups had different influences on the inhibition. However, there was no apparent regularity for the effects of variation with a mono-substituent on position 4 of phenyl ring. Furthermore, compounds **13** to **14**, **24** to **25** and **38** to **43** with double substituents on benzene ring displayed much stronger anti-leishmanial activity against *L. donovani*. However, positions of the substituents had apparent influences on the inhibition behavior. For example, *N*-2-methyl-3-nitro-substituted compound (**42**) exhibited the highest anti-leishmanial activity (IC_50_ < 0.0128 μg/mL). And **13**, **14**, **39**, **40**, and **41** also showed very strong anti-leishmanial activity (IC_50_ ranged from 0.08–0.13 μg/mL). In spite of these factors, steric hindrances might affect anti-leishmanial activity. From the results in Table 1, it can be concluded that the logP value (predicted by Chemoffice 2014) is an important parameter influencing the anti-leishmanial activities against *L. donovani*. However, there was no apparent regularity between the IC_50_ values and log P values on the whole. 

## 3. Experimental Section

### 3.1. Chemistry General Details

Starting materials were obtained from Aldrich and used as received. Melting points (MP) were measured with a WRS-1A melting point apparatus, and were uncorrected. ^1^H-NMR spectra were recorded on a Bruker AVANCE III 500 spectrometer (Bruker, London, UK) at 500 MHz using tetramethylsilane (TMS) as an internal standard. Electrospray ionization-mass spectra (EIMS) were measured on a mass spectrometer (Thermo Fisher Scientific, LCQ/ADVANTAGE, 81 Wyman Street, Waltham, MA, USA). IR spectrum was recorded in KBr pellets on a Nicolet 6700 infrared spectrophotometer (Thermo Fisher Scientific, 81 Wyman Street, Waltham, MA, USA).

The structures of all the compounds were determined by IR, EI–MS, ^1^H-NMR. Note that compounds **1**–**12**, **17**, **21**, **22**, **28**–**37** had been previously reported [27,33,39,40,41,42,43], which were coincident with the previous report by EI MS and ^1^H-NMR. Physical and spectroscopic data of the other compounds were shown in the supplementary material.

### 3.2. Biology

In vitro anti-leishmanial assay. The antileishmanial activity of the compounds was tested in vitro against a culture of *L. donovani* promastigotes [44]. The promastigotes were grown in RPMI 1640 medium supplemented with 10% fetal calf serum (Gibco Chem. Co., 81 Wyman Street, Waltham, MA, USA) at 26 °C. A three day old culture was diluted to 5 × 10^5^ promastigotes/mL. Drug dilutions were prepared directly in cell suspension in 96-well plates. Plates were incubated at 26 °C for 48 h and the growth of *Leishmania* promastigotes was determined by the Alamar blue assay as described earlier [45]. Standard fluorescence was measured on a Fluostar Galaxy plate reader (BMG Lab Technologies, Offenburg, Germany) at an excitation wavelength of 544 nm and an emission wavelength of 590 nm. Pentamidine and amphotericin B were used as the standard anti-leishmanial agents. IC_50_ values were computed from dose-response curves as above. The tested compounds were diluted with six concentrations (from 40–0.0128 μg/mL). Cytotoxicity assay. The in vitro cytotoxicity was determined against human monocytic leukemia cells (THP1) with a simple colorimetric method using the dye Alamar Blue [45]. THP1 suspensions were grown in RPMI-1640 medium supplemented with 10% FBS, 2 mM glutamine, 50 μg/mL gentamicin and 0.0025 mg/L of amphotericin B (Sigma) at 37 °C in a 5% CO_2_ atmosphere. Cells were grown to a density between 0.2 and 1 × 10^6^ cells/mL. Culture medium was replaced every 2–3 days with fresh growth medium. DMSO was used as the solvent, and the test compounds was with six concentrations from 10–0.0032 μg/mL.

## 4. Conclusions

In summary, a series of maleimides have been synthesized and evaluated for inhibitory activities against *L. donovani*. All compounds exhibited obvious anti-leishmanial activities, especially with compounds **16** and **42** showing extraordinary potency in an in vitro test and low cytotoxicity. The anti-leishmanial activities of the two compounds were 10 times better than that of amphotericin B. Therefore, further preclinical studies of **16** and **42** aimed at leishmaniasis are important for the therapy of this neglected disease. The cytotoxicity of these compounds was low, with nearly no toxicity (>10 μg/mL). Thus, compounds **16** and **42** are promising candidates for visceral leishmaniasis. Among the tested compounds, there were 10 maleimides with superior anti-leishmanial activities to amphotericin B, and 32 maleimides with superior anti-leishmanial activities to pentamidine. The SAR study showed that 3,4-non-substituted maleimides displayed the strongest anti-leishmanial activities compared to those 3-methyl-maleimides and 3,4-dichloro-maleimides. When the length of the alkyl side chain (*N*-alkyl and *N*-phenylalkyl) increased, the anti-leishmanial activities decreased significantly. There was no obvious regularity for the influences of variation with a mono-substituent on phenyl ring position 4. And the position of the substituent had an obvious influence on the inhibition behavior. As to cytotoxicity, 3, 4-dichloro-maleimides were least cytotoxic compared to 3-methyl-maleimides and 3,4-non-substituted maleimides.

## Figures and Tables

**Table 1:** Anti-leishmanial activities and cytotoxicity of maleimides **1**–**45**, pentamidine and amphotericin B.

Compound	*L. donvani* IC_50_ (μg/mL)	THP1 CC_50_ (μg/mL)	SI	LogP
**1**	0.08	>10	>125.0	0.72
**2**	0.36	>10	>27.8	1.14
**3**	0.11	>10	>90.9	1.56
**4**	0.27	>10	>37.0	2.39
**5**	2.21	>10	>4.5	4.06
**6**	0.08	4.56	57.0	1.21
**7**	0.10	4.13	41.3	1.49
**8**	0.11	8.25	75.0	1.91
**9**	0.24	>10	>41.7	1.14
**10**	0.15	>10	>66.7	1.63
**11**	0.32	>10	>31.3	1.30
**12**	1.02	>10	>9.8	1.70
**13**	0.10	3.65	36.5	2.12
**14**	0.08	3.89	48.6	2.95
**15**	0.21	>10	>23.2	1.62
**16**	<0.0128	>10	>781.3	0.70
**17**	0.26	>10	>38.5	1.03
**18**	0.58	7.64	13.2	1.07
**19**	0.81	5.89	7.3	1.91
**20**	0.96	8.10	8.4	2.74
**21**	0.58	7.94	13.7	1.56
**22**	1.22	5.99	4.9	1.84
**23**	13.17	>10	>0.8	2.47
**24**	0.55	4.39	8.0	3.30
**25**	0.34	0.80	2.4	2.61
**26**	0.64	>10	>15.6	1.05
**27**	0.89	7.15	8.0	1.38
**28**	0.47	>10	>21.3	0.80
**29**	0.65	>10	>15.4	1.22
**30**	0.49	>10	>20.4	1.63
**31**	0.48	>10	>20.8	2.47
**32**	1.64	>10	>6.1	1.57
**33**	0.40	>10	>25.0	1.99
**34**	0.76	>10	>13.2	1.22
**35**	0.66	>10	>15.2	1.71
**36**	0.36	>10	>27.8	1.38
**37**	0.59	>10	>16.9	1.78
**38**	0.45	>10	>22.2	2.19
**39**	0.13	>10	>76.9	3.03
**40**	0.13	>10	>76.9	2.27
**41**	0.11	>10	>90.9	2.27
**42**	<0.0128	>10	>781.3	1.70
**43**	0.99	>10	>10.1	2.34
**44**	0.43	>10	>23.3	0.78
**45**	0.26	>10	>38.5	1.10
pentamidine	0.64	ND	-	2.84
amphotericin B	0.12	ND	-	2.30

## Supplementary Materials

The following are available online, the structure data of all maleimides and the biological methods in detail.
