# Supplementary material for: Anti-Leishmanial and Cytotoxic Activities of a Series of Maleimides: Synthesis, Biological Evaluation and Structure-Activity Relationship
Source: Molecules. 2018 Nov 5;23(11):2878. doi: 10.3390/molecules23112878 (PMC6278306; doi:10.3390/molecules23112878)
Supplement: Supplementary file 1 [file molecules-23-02878-s001.pdf]

Supplementary Material

Physical and spectroscopic data of the studied compounds were shown as following:

***N*-butyl-maleimide (1):** CAS Registry Number 2973-09-3, Yield 58.7%. Yellow oil. ^{1}H NMR (500 MHz, $CDCl_3$) δ 6.71 (s, 2H), 3.61 (t, *J*=7.3Hz, 2H), 1.65-1.59 (m, 2H), 1.33 (dq, *J*=14.8,7.4 Hz, 2H), 0.95(t, *J=7.4* Hz, 3H). ESI-MS m/z(%): 153.1 (31) $[M]^{+}$, 110.0 (100), 82.1 (30), 54.1 (16), 41.1 (5).

***N*-amyl-maleimide (2):** CAS Registry Number 19775-00-9, Yield 53.4%. Light yellow oil. ^{1}H NMR (500 MHz, $CDCl_3$) δ 6.71 (s, 2H), 3.64-3.57 (m, 2H), 1.63 (dt, *J*=14.9,7.5Hz, 2H), 1.40-1.24 (m,4H), 0.91 (t, *J*=7.2Hz, 3H). ESI-MS m/z (%): 167.1 (49) $[M]^{+}$, 138.1 (8), 124.1 (15), 110.0 (100), 82.1 (31), 69.1 (8), 54.1 (16), 41.1 (14).

***N*-hexyl-maleimide (3):** CAS Registry Number 17450-29-2, Yield 49.2%. Oil. ^{1}H NMR (500 MHz, $CDCl_3$) δ 6.71 (s, 2H), 3.63-3.58 (m,2H), 1.62 (dd, *J*=13.9,7.2Hz, 2H), 1.30(s, 6H), 0.89 (t, *J=6.6* Hz, 3H). ESI-MS m/z (%): 181.1 (34) $[M]^{+}$, 152.1 (8), 138.1 (14), 124.1 (21), 110.0 (100), 82.0 (34), 55.1 (22), 41.1 (14).

***N*-octyl-maleimide (4):** CAS Registry Number 4080-76-6, Yield 57.6%. Light yellow plate crystals. ^{1}H NMR (500 MHz, $CDCl_3$) δ6.71 (s, 2H), 3.63-3.57 (m, 2H), 1.65-1.58 (m,2H), 1.30 (td, *J*=12.9,5.6Hz, 10H), 0.89 (t, *J=7.0*Hz, 3H). ESI-MS m/z (%): 209.2 (50) $[M]^{+}$, 138.1 (21), 110.0 (100), 82.0 (32), 55.1 (24), 39.1 (22).

***N*-dodecyl-maleimide (5):** CAS Registry Number 17616-03-4, Yield 59.3%. White plate crystals. ^{1}H NMR (500 MHz, $CDCl_3$) δ6.71 (s, 2H), 3.64-3.56(m, 2H),1.66-1.58 (m, 2H), 1.35-1.24 (m, 18H), 0.89(t, *J*=7.0Hz, 3H). ESI-MS m/z (%): 265.1 (100) $[M]^{+}$, 208.2 (5), 166.1 (8), 138.1 (18), 110.0 (72), 82.0 (18), 55.1 (18), 33.1 (17).

***N*-benzyl-maleimide (6):** CAS Registry Number 1631-26-1, Yield 79.4%. Light white power crystals. ^{1}H NMR (500 MHz, $CDCl_3$) δ7.38-7.35 (m, 2H), 7.35-7.31 (m, 2H), 7.29 (dq, *J*=3.9,1.7Hz, 1H), 6.72 (d, *J*=5.0Hz, 2H), 4.69 (s, 2H). ESI-MS m/z (%): 187.1 (100) $[M]^+$, 173.0 (7), 159.1 (18), 130.1 (49), 104.1 (56), 78.1 (22), 54.1 (16), 39.1 (7).

***N*-(β-phenylethyl)-maleimide (7):** CAS Registry Number 6943-90-4, Yield 73.2%. Light white power crystals. ^{1}H NMR (500MHz, $CDCl_3$) δ7.33-7.27 (m, 2H), 7.23 (ddd, *J*=13.1,6.8,4.1Hz, 3H), 6.66 (d, *J*=5.1Hz, 2H), 3.81-3.75 (m,2H), 2.94-2.89 (m,2H). ESI-MS m/z (%): 201.1 (44) $[M]^+$, 104.18 (100), 82.1 (10), 65.1 (10), 39.1(5).

***N*-(3-phenpropyl)-maleimide (8):** CAS Registry Number 28537-62-4, Yield 71.2%. White power crystals. ^{1}H NMR (500MHz, $CDCl_3$) δ7.31-7.27 (m, 2H), 7.20 (t, *J*=3.4Hz, 2H), 7.19-7.17 (m, 1H), 6.69-6.65 (m, 2H), 3.59(t, *J*=7.2Hz, 2H), 2.68-2.61 (m, 2H), 1.96 (dt, *J*=14.9,7.6 Hz, 2H). ESI-MS m/z (%): 215.1 (54) $[M]^+$, 117.1 (100), 91.1 (32), 65.1 (12), 39.1 (3).

***N*-phenyl-maleimide(9):** CAS Registry Number 941-69-5, Yield 69.5%. Yellow needle crystals. ^{1}HNMR (500MHz, $CDCl_3$) δ7.38-7.35 (m, 2H), 7.35-7.31(m, 2H), 7.29 (dq, *J*=3.9, 1.7Hz, 1H), 6.72 (d, *J*=5.0Hz, 2H). ESI-MSm/z(%):173.1(100) $[M]^+$, 145.1 (6), 129.1 (27), 116.1 (16), 103.1 (17), 90.1 (14), 77.1 (8), 54.0 (15), 39.1 (3).

***N*-(4-tolyl)-maleimide(10):** CAS Registry Number 1631-28-3, Yield 71.5%. Yellow powder. ^{1}HNMR (500MHz, $CDCl_3$) δ7.28 (d, *J=3.0*Hz,2H), 7.25-7.19(m, 2H), 6.48 (q,*J=1.8*Hz, 1H), 2.39 (s, 3H), 2.19 (d, *J=1.8*Hz,3H). ESI-MS m/z (%): 187.1(100) $[M]^+$,172.0 (6), 158.1 (10), 130.1 (27), 104.1 (11), 77.1 (10), 54.1(11), 39.1 (3).

***N*-(4-fluorophenyl)-maleimide(11):** CAS Registry Number 6633-22-3, Yield 71.8%. Yellow powder. ^{1}HNMR (500MHz, $CDCl_3$) δ7.36-7.32 (m,2H), 7.21-7.15(m,2H), 6.88 (s,2H) .ESI-

MSm/z(%): 191.0 (100)[M]$^+$, 163.0(5), 147.1(17), 121.0(27), 82.0(11), 54.0(17).

***N*-(4-chlorophenyl)-maleimide(12):** CAS Registry Number 1631-29-4, Yield 72.5%. Yellow powder. ^{1}HNMR (500MHz, $CDCl_3$) δ7.48-7.44 (m,2H), 7.36-7.30 (m,2H), 6.88 (s, 2H). ESI-MS m/z (%): 207(100) [M]$^+$, 179.0(4), 163.0(16), 137.0(25), 116.0(8), 90.1(16), 74.0(7), 54.0(24), 32.1(4).

***N*-(2, 6-Dimethylphenyl)-maleimide (13):** CAS Registry Number 1206-49-1, Yield 70.6%. Light yellow crystals. Mp 188.8-190.4 ℃. IR (KBr) cm^{-1}: 3465, 3088, 2932, 1714, 1576, 1453, 1386, 1090, 852, 720, 703, 524. ^{1}H NMR (500 MHz, $CDCl_3$) δ 7.25 (d, *J* = 7.6 Hz, 1H), 7.16 (d, *J* = 7.6 Hz, 2H), 6.90 (s, 2H), 2.13 (s, 6H). EI-MS m/z (%): 201.1(100) [M]$^+$, 183.1(98), 144.1(40), 118.1(15), 91.1(23), 76.1(18), 54(15), 39.1(8).

***N*-(2, 6-Diethylphenyl)-maleimide (14):** CAS Registry Number 38167-72-5, Yield 81.2%. Brown powder crystals. Mp 192.8-194.1 ℃. IR (KBr) cm^{-1}: 3469, 3091, 2934, 1719, 1570, 1453, 1386, 1090, 853, 722, 700, 524. ^{1}H NMR (500 MHz, $CDCl_3$) δ 7.38 (t, *J* = 7.7 Hz, 1H), 7.22 (d, *J* = 7.7 Hz, 2H), 6.90 (s, 2H), 2.42 (q, *J* = 7.6 Hz, 4H), 1.15 (dd, *J* = 9.5, 5.7 Hz, 6H). EI-MS m/z (%): 229.1 (100) [M]$^+$, 196.1 (48), 158.1 (30), 132.1(42), 115.1(19), 77.1(20), 54.1(18), 37.0 (7).

***N*-(3, 4, 5-Trifluorophenyl)-maleimide (15):** CAS Registry Number 1188945-12-1, Yield 71.8%. White powder crystals. Mp 153.4-165.6 ℃. IR (KBr) cm^{-1}: 3478, 3081, 1718, 1627, 1521, 1458, 1323, 1231, 1097, 1040, 852, 723, 690, 529. ^{1}H NMR (500 MHz, $CDCl_3$) δ 7.20 – 7.13 (m, 2H) 6.89 (s, 2H). EI-MS m/z (%): 227.0 (100) [M]$^+$, 183.0 (15), 157.0 (20), 118.0 (7), 95.0 (4), 75.0 (4), 54.0 (18).

***N-iso*-Butyl-maleimide (16):** CAS Registry Number 4120-68-7, Yield 70.1%. Light yellow plate crystals. Mp 52.8-53.9 ℃. IR (KBr) cm^{-1}: 3457, 2963, 2929, 2874, 1711, 1439, 1408, 1375, 1059, 734, 521. ^{1}H NMR (500 MHz, $CDCl_3$) δ 6.71 (s, 2H), 3.34 (d, J = 7.4 Hz, 2H), 2.02 (dp, J = 14.0, 6.9 Hz, 1H), 0.90 (d, J = 6.7 Hz, 6H). EI-MS m/z (%): 153.1 (49) $[M]^+$, 110.0 (100), 82.0 (38), 54.1 (21), 41.1 (11).

***N*-cyclohexyl-maleimide (17):** CAS Registry Number 1631-25-0, Yield 68.5%. Light yellow power crystals. ^{1}H NMR (500 MHz, $CDCl_3$) δ6.63 (s, 2H), 3.95-3.88 (m, 1H), 1.26 (tt, *J*=2.0,1.0Hz, 2H), 1.23 (d, *J*=2.1Hz, 2H), 1.20-1.12 (m, 6H). ESI-MS m/z (%): 179.1 (43) $[M]^+$, 136.0 (56), 99.0 (100), 81.1 (44), 54.1 (33), 39.1 (20).

***N*-Pentyl-3-methylmaleimide (18):** CAS Registry Number 143659-00-1, Yield 79.1%. Light yellow oil. IR (KBr) cm^{-1}: 3452, 2952, 2930, 2862, 1701, 1441, 1403, 1373, 1061, 734, 528. ^{1}H NMR (500 MHz, $CDCl_3$) δ 6.31 (q, *J* = 1.7 Hz, 1H), 3.50 – 3.47 (m, 2H), 2.08 (d, *J* = 1.8 Hz, 3H), 1.61 – 1.54 (m, 2H), 1.37 – 1.29 (m, 2H), 1.29 – 1.22 (m, 2H), 0.89 (t, *J* = 7.2 Hz, 3H). EI-MS m/z (%): 181.1(38) $[M]^+$, 138.1(18), 124.1 (100), 96.1 (15), 70.1 (12), 56.1 (15), 39.1(15).

***N*-Hexyl-3-methylmaleimide (19):** CAS Registry Number 73383-84-3, Yield 79.6%. Light yellow oil. IR (KBr) cm^{-1}: 3459, 2952, 2931, 2859, 1702, 1442, 1407, 1377, 1064, 734, 52o. ^{1}H NMR (500 MHz, $CDCl_3$) δ 6.30 (q, *J* = 1.8 Hz, 1H), 3.50–3.46 (m, 2H), 2.08 (d, *J* = 1.8 Hz, 3H), 1.56 (p, *J* = 7.3 Hz, 2H), 1.31 – 1.25 (m, 6H), 0.87 (t, *J* = 6.8 Hz, 3H). EI-MS m/z (%): 193.1(38) $[M]^+$, 150.1(50), 112.1 (100), 96.1 (3514), 81.1 (38), 67.1 (42), 53.1 (22), 39.1 (47).

***N*-Octyl-3-methylmaleimide (20):** CAS Registry Number 100522-72-3, Yield 77.6%. Light yellow oil. IR (KBr) cm^{-1}: 3346, 2929, 2851, 1704, 1440, 1406, 1372, 1072, 733, 528. ^{1}H NMR (500 MHz, $CDCl_3$) δ 6.30 (q, *J* = 1.8 Hz, 1H), 3.50–3.46 (m, 2H), 2.08 (d, *J* = 1.8

Hz, 3H), 1.56 (p, *J* = 7.3 Hz, 2H), 1.31 – 1.25 (m, 6H), 0.87 (t, *J* = 6.8 Hz, 3H). EI-MS m/z (%): 223.2 (47) $[M]^+$, 180.1 (16), 152.1 (11), 124.1 (100), 96.1 (18), 69.1 (16), 41.1 (18).

***N*-benzyl-3-methylmaleimide (21):** CAS Registry Number 73383-82-1, Yield 73.2%. Oil. ^{1}H NMR (500 MHz, $CDCl_3$) δ7.38-7.34(m, 2H), 7.34-7.30 (m, 2H), 7.30-7.26 (m, 1H), 6.34 (q, *J*=1.8Hz, 1H), 4.67 (s, 2H), 2.09 (d, *J*=1.8Hz, 3H). ESI-MS m/z(%): 201.1 (100) $[M]^+$, 172.1 (30), 144.1 (25), 104.1 (45), 78.1 (20), 63.1 (12), 39.1 (17).

***N*-(β-phenylethyl)-3-methymaleimide (22):** CAS Registry Number 140369-62-6, Yield 76.4%. Light yellow plate crystals. ^{1}H NMR (500MHz, $CDCl_3$) δ7.30 (t, *J*=7.5*Hz*, 2H), 7.24 (d, *J*=6.4Hz, 2H), 7.21 (s, 1H), 6.30 (d, *J*=1.7Hz, 1H), 3.78-3.72 (m, 2H), 2.93-2.88 (m, 2H), 2.07 (d, *J*=1.6Hz, 3H). ESI-MS m/z (%): 215.1 (98) $[M]^+$, 124.0 (81), 104.1 (100), 56.1 (8), 39.1 (7).

***N*-(2, 6-Dimethylphenyl)-3-methylmaleimide (23):** CAS Registry Number 117659-65-1, Yield 75.7%. Brown powder crystals. Mp 190.8-192.0 ℃. IR (KBr) cm^{-1}: 3460, 3099, 2922, 1711, 1570, 1459, 1380, 1097, 858, 728, 701, 524. ^{1}H NMR (500 MHz, $CDCl_3$) δ 7.27 – 7.22 (m, 1H), 7.15 (d, *J* = 7.6 Hz, 2H), 6.52 (q, *J* = 1.8 Hz, 1H), 2.21 (d, *J* = 1.8 Hz, 3H), 2.13 (s, 6H). EI-MS m/z (%): 215.1(100)$[M]^+$, 197.1(61), 168.1(22), 144.1 (31), 131.1 (17), 91. 1(20) , 77.1 (14), 39.1 (18).

***N*-(2, 6-Diethylphenyl))-3-methylmaleimide (24):** CAS Registry Number 58100-55-3, Yield 79.7%. Brown granular crystals. Mp 195.8-197.3 ℃. IR (KBr) cm^{-1}: 3458, 3081, 2925, 1714, 1575, 1452, 1384, 1090, 857, 720, 704, 527. ^{1}H NMR (500 MHz, $CDCl_3$) δ 7.36 (t, *J* = 7.7 Hz, 1H), 7.21 (d, *J* = 7.7 Hz, 2H), 6.53 (q, *J* = 1.7 Hz, 1H), 2.42 (q, *J* = 7.6 Hz, 4H), 2.21 (d, *J* = 1.8 Hz, 3H), 1.15 (t, *J* = 7.6 Hz, 6H). EI-MS m/z (%):243.1(100)$[M]^+$, 214.1(34), 198.1(31), 186.1 (14), 172.1(13), 158.1(14) , 132.1(28), 117.1(27), 91.1(14), 77.1(14), 39.1(15).

***N*-(3,5-Dichlorophenyl)-3-methylmaleimide (25):** CAS Registry Number 27035-07-0, Yield 80.5%. Brown crystals. Mp 170.8-173.2 ℃. IR (KBr) cm^{-1}: 3469, 3091, 2924, 1712, 1573, 1450, 1387, 1092, 854, 724, 701, 525. ^1H NMR (500 MHz, $CDCl_3$) δ7.43 (t, *J* = 1.8 Hz, 1H), 7.36 (d, *J* = 1.8 Hz, 2H), 6.53 (q, *J* = 1.7 Hz, 1H), 2.21 (d, *J* = 1.8 Hz, 3H). EIMS m/z (%): 255.0(100)$[M]^+$, 211.0(14), 187.1(15), 124.0 (16), 68.1(27), 40.1(20).

***N*-*iso*-Butyl-3-methylmaleimide (26):** CAS Registry Number 15760-40-4, Yield 70.8%. Light yellow oil. IR (KBr) cm^{-1}: 3449, 2959, 2929, 2872, 1721, 1439, 1408, 1375, 1059, 734, 522. ^1H NMR (500 MHz, $CDCl_3$) δ 6.31 (q, *J* = 1.8 Hz, 1H), 3.30 (d, *J* = 7.4 Hz, 2H), 2.08 (d, *J* = 1.9 Hz, 3H), 2.00 (td, *J* = 13.9, 6.9 Hz, 1H), 0.87 (d, *J* = 6.7 Hz, 6H). EI-MS m/z (%): 167.1 (35) $[M]^+$, 134.1 (100), 96.1 (12), 56.1 (15), 39.1 (15).

***N*-Cyclohexyl-3-methylmaleimide (27):** CAS Registry Number 91247-10-8, Yield 70.3%. Light yellow power crystals. Mp 65.6-66.5℃. IR (KBr) cm^{-1}: 3449, 2932, 2862, 1703, 1400, 1381, 1089, 733, 526. ^1H NMR (500 MHz, $CDCl_3$) δ 6.26 (q, *J* = 1.8 Hz, 1H), 3.88 (tt, *J* = 12.3, 3.9 Hz, 1H), 2.05 (d, *J* = 1.8 Hz, 3H), 2.04 (qd, *J* = 12.5, 3.4 Hz, 2H), 1.86 (dd, *J* = 16.2, 2.6 Hz, 2H), 1.71–1.65 (m, 2H), 1.38–1.17 (m, 4H). EI-MS m/z (%): 193.1 (43) $[M]^+$, 150.1 (53), 112.0 (100), 96.1(38), 81.1 (41), 67.1 (45), 53.1 (22), 39.1 (45).

***N*-butyl-3,4-dichloromaleimide (28):** CAS Registry Number 20198-79-2, Yield 82.5%. Light red power crystals. ^1H NMR (500MHz, $CDCl_3$) δ3.61 (t, *J*=7.3Hz, 2H), 1.65-1.59 (m, 2H), 1.33(dq, *J*=14.8,7.4Hz, 2H), 0.95 (t,*J*=7.4Hz, 3H). ESI-MS m/z(%): 221.0 (25) $[M]^+$, 177.9 (100), 149.9 (7), 116.0 (13), 87.0 (45), 56.1 (34), 39.1 (16).

***N*-amyl-3,4-dichloromaleimide (29):** CAS Registry Number 81878-26-4, Yield 80.8%. Oil. ^1H NMR (500MHz, $CDCl_3$) δ3.64-3.57 (m, 2H), 1.63 (dt, *J*=14.9, 7.5Hz, 2H), 1.40-1.24 (m,4H),

0.91 (t, *J*=7.2Hz, 3H). ESI-MS m/z (%): 235.0 (22) $[M]^+$, 178.0 (100), 136.1 (11), 116.0 (17), 87.0 (44), 69.1 (25), 41.1 (23).

***N*-hexyl-3,4-dichloromaleimide (30):** CAS Registry Number 52106-40-8, Yield 81.1%. Red brown crystals. ^1H NMR (500 MHz, $CDCl_3$) δ3.61 (t, *J*=7.3Hz, 2H), 1.65-1.59 (m, 2H), 1.33 (dq, *J*=14.8, 7.4Hz,2H), 0.95(t, *J*=7.4Hz, 3H). ESI-MS m/z (%): 249.0 (34) $[M]^+$, 214.1 (9), 178.0 (100), 150.0 (16), 116.0 (9), 87.0 (28), 108 (6), 55.1 (25), 29.1 (7).

***N*-octyl-3,4-dichloromaleimide (31):** CAS Registry Number 51302-94-4, Yield 85.2%. Light yellow plate crystals. ^1H NMR (500 MHz, $CDCl_3$) δ3.63-3.57 (m, 2H), 1.65-1.58 (m, 2H), 1.30 (td, *J*=12.9,5.6Hz, 10H), 0.89 (t, *J*=7.0Hz, 3H). ESI-MS m/z (%): 277.1 (21) $[M]^+$, 242.1 (10), 214.1 (75), 178.0 (100), 116.0 (6), 69.1 (98), 41.1 (68).

***N*-(2-phenylethyl)-3,4-dichloromaleimide (32):** CAS Registry Number 3116-49-2, Yield 80.7%. Light yellow power crystals. ^1H NMR (500 MHz, $CDCl_3$) δ7.32 (dd, *J*=10.1, 4.5Hz, 2H), 7.26 (td, *J*=7.0,3.2Hz,1H), 7.23-7.20 (m,2H), 3.86 (dd, *J*=8.4,6.9Hz, 2H), 2.99-2.91 (m, 2H). ESI-MS m/z (%): 269.0 (15) $[M]^+$, 180.0 (17), 104.1 (100), 65.1 (16), 39.1 (8).

***N*-(3-phenpropyl)-3,4-dichloromaleimide (33):** CAS Registry Number 92751-36-5, Yield 82.3%. Light yellow plate crystals. ^1HNMR (500 MHz, $CDCl_3$) δ7.28 (d, *J*=15.0Hz, 2H), 7.18 (dd, *J*=16.5, 7.3Hz, 3H), 3.67 (t, *J*=7.0Hz, 2H), 2.67 (t, *J*=7.5Hz, 2H), 2.08-1.99 (m, 2H). ESI-MS m/z (%): 283.0 (8) $[M]^+$, 180.0 (23), 117.1 (100), 91.1 (62), 65.1 (21), 39.1 (8).

***N*-phenyl-3,4-dichloromaleimide (34):** CAS Registry Number 3876-05-9, Yield 73.5%. Light yellow crystals. ^1H NMR (500 MHz, $CDCl_3$) δ7.53-7.48 (m, 2H), 7.43 (dd, *J*=8.5,6.4Hz, 1H), 7.38-7.33 (m, 2H). ESI-MS m/z (%): 241.0 (100) $[M]^+$, 162 (72), 119.0 (60), 87.0 (52), 64.1 (21), 39.1 (7).

***N*-(4-tolyl)-3,4-dichloromaleimide (35):** CAS Registry Number 29244-55-1, Yield 79.5%. Red brown crystals. ^{1}H NMR (500 MHz, $CDCl_3$) δ7.30 (d, *J*=8.2Hz, 2H), 7.24-7.20 (m, 2H), 2.41 (s, 3H). ESI-MS m/z (%): 255.0 (100) $[M]^+$, 226.0 (10), 192.0 (9), 176.1 (28), 133.1 (39), 104.0 (17), 133.1 (23), 104.0 (18), 87.0 (24), 65.0 (4), 51.1 (6), 39.1 (4).

***N*-(4-fluorophenyl)-3,4-dichloromaleimide (36):** CAS Registry Number 41205-21-4, Yield 90.1%. Light yellow crystals. ^{1}H NMR (500 MHz, $CDCl_3$) δ7.37-7.32 (m, 2H), 7.22-7.17 (m, 2H). ESI-MS m/z (%): 259 (100) $[M]^+$, 196.0 (5), 180 (48), 137.0 (49), 109.0 (30), 87.0 (43), 75.1 (5), 57.0 (5), 44.0 (7), 32.1 (5).

***N*-(4-chlorophenyl)-3,4-dichloromaleimide (37):** CAS Registry Number 29236-09-7, Yield 83.5%. Yellow power crystals. ^{1}H NMR (500 MHz, $CDCl_3$) δ7.50-7.45 (m, 2H), 7.35-7.30 (m, 2H). ESI-MS m/z (%):276.5 (100) $[M]^+$, 196.0 (45), 153.0 (63), 124.9 (25), 95.9 (14), 87.0 (46), 63.0 (18), 50.1 (4), 36.0 (4).

***N*-(2, 6-Dimethylphenyl)-3, 4-dichloromaleimide (38):** CAS Registry Number 35740-43-3, Yield 85.8%. Light yellow power crystals. Mp 200.8-201.6℃. IR (KBr) cm^{-1}: 3469, 3084, 2923, 1706, 1574, 1464, 1389, 1082, 859, 726, 704, 527. ^{1}H NMR (500 MHz, $CDCl_3$) δ 7.30 (d, *J* = 7.6 Hz, 1H), 7.18 (d, *J* = 7.6 Hz, 2H), 2.15 (s, 6H). EI-MS m/z (%): 269.0 (100) $[M]^+$, 251.0 (42), 226.0 (13), 206.1 (16), 190.0(63), 146.1(16), 132.1(9), 118.1(16), 105.1(19), 87.0(25), 77.1(18), 65.1(11), 51.1(10), 39.1(10).

***N*-(2, 6-Diethylphenyl)-3, 4-dichloromaleimide (39):** CAS Registry Number 56746-18-0, Yield 87.3%. Brown power crystals. Mp 204.1-205.8 ℃. IR (KBr) cm^{-1}: 3472, 3098, 2928, 1713, 1579, 1458, 1389, 1098, 859, 726, 706, 529. ^{1}H NMR (500 MHz, $CDCl_3$) δ7.41 (t, *J* = 7.7 Hz, 1H), 7.23 (d, *J*=7.7 Hz, 2H), 2.43 (q, *J*=7.6 Hz, 4H), 1.17 (t, *J* = 7.6 Hz, 6H). EI-MS m/z (%):

181 (32). EIMS m/z (%): 297.1 (100) $[M]^+$, 282.0(29), 268.0(32),250.0(8), 232.0(17), 132.1(75), 117.1 (50), 105.1(8), 87.0(17), 65.1(4), 39.1(3).

***N*-(2-Methyl-3-chloro-phenyl)-3, 4-dichloromaleimide (40):** CAS Registry Number 58623-32-8, Yield 78.2%. Light yellow power crystals. Mp 162.3-164.1℃. IR (KBr) cm^{-1}: 3473, 3090, 1706, 1539, 1382, 1361, 1090, 730, 715, 522. ^1H NMR (500 MHz, $CDCl_3$) δ 7.52 (dd, *J* = 8.1, 0.7 Hz, 1H), 7.27 (t, *J* = 8.0 Hz, 1H), 7.08 (dd, *J* = 7.9, 0.6 Hz, 1H), 2.22 (s, 3H). EI-MS m/z (%): 289.0 (100) $[M]^+$, 271.0 (60), 254.0 (27), 226.0 (33), 210.0 (50), 166.0 (20), 132.0(17), 87.0 (50), 77.1 (23), 63.0 (10), 51.1(14), 39.1(8).

***N*-(2-Methyl-5-chloro-phenyl)-3, 4-dichloromaleimide (41):** CAS Registry Number 58100-79-1, Yield 77.4%. Yellow power crystals. Mp 160.1-161.4 ℃. IR (KBr) cm^{-1}: 3460, 3084, 1712, 1537, 1380, 1368, 1090, 734, 715, 528. ^1H NMR (500 MHz, $CDCl_3$) δ 7.38 (dd, *J* = 8.3, 2.1 Hz, 1H), 7.30 (d, *J* = 8.3 Hz, 1H), 7.17 (d, *J* = 2.1 Hz, 1H), 2.17 (s, 3H). EI-MS m/z (%): 289.0 (100) $[M]^+$, 271.0 (50), 254.0 (15), 226.0 (49), 210.0 (38), 166.0 (34), 132.1 (25), 87.0 (57), 63.0 (10), 51.1 (15).

***N*-(2-Methyl-3-nitro-phenyl)-3, 4-dichloromaleimide (42):** CAS Registry Number 1439404-79-1, Yield 83.1%. Light yellow power crystals. Mp 168.8-169.9℃. IR (KBr) cm^{-1}: 3468, 3090, 1701, 1538, 1384, 1360, 1099, 730, 715, 527. ^1H NMR (500 MHz, $CDCl_3$) δ 8.03 (d, *J* = 8.2 Hz, 1H), 7.53 – 7.48 (m, 1H),7.43 (dd, *J* = 7.9, 1.1 Hz, 1H), 2.35 (s, 3H). EI-MS m/z (%): 300.0 (6) $[M]^+$, 283.0 (100), 255.0 (46), 191.0 (60), 161.0 (23), 148.0 (20), 106.0 (21), 87 (86), 77.1 (69), 63.0 (18), 51.1(34), 39.1(9).

***N*-(3, 5-Dichlorophenyl)-3, 4-dichloromaleimide (43):** CAS Registry Number 84174-90-3, Yield 90.3%. Yellow crystals. Mp 190.2-191.4 ℃. IR (KBr) cm^{-1}: 3463, 3094, 2926, 1716,

1577, 1454, 1387, 1092, 854, 724, 702, 521. ^{1}H NMR (500 MHz, $CDCl_3$) δ 7.43 (t, *J* = 1.8 Hz, 1H), 7.36 (d, *J* = 1.8 Hz, 2H). EI-MS m/z (%): 309.0 (100) $[M]^+$, 229.9(27), 186.9(14), 124.0 (28), 87.0 (34).

***N-iso*-Butyl-3, 4-dichloromaleimide (44):** CAS Registry Number 52106-39-5, Yield 70.8%. Light yellow crystals crystals. Mp 63.8-64.5 ℃. IR (KBr) cm^{-1}: 3462, 2957, 2925, 2874, 1711, 1439, 1408, 1375, 1059, 734, 525. ^{1}H NMR (500 MHz, $CDCl_3$) δ 3.43 (d, *J* = 7.4 Hz, 2H), 2.04 (dp, *J* = 13.9, 6.9 Hz, 1H), 0.92 (d, *J* = 6.7 Hz, 6H). EI-MS m/z (%): 221.0 (38) $[M]^+$, 178.0 (100), 151.0 (8), 116.0 (28), 87.0(58), 56.1(72), 39.1 (38).

***N*-Cyclohexyl-3, 4-dichloromaleimide (45):** CAS Registry Number 50343-26-5, Yield 71.2%. Brown power crystals. Mp 67.2-68.5 ℃. IR (KBr) cm^{-1}: 3439, 2925, 2862, 1720, 1398, 1380, 1089, 733, 528. ^{1}H NMR (500 MHz, $CDCl_3$) δ 4.00 (tt, *J* = 12.4, 3.9 Hz, 1H), 2.04 (qd, *J* = 12.5, 3.4 Hz, 2H), 1.86 (dd, *J* = 16.2, 2.6 Hz, 2H), 1.71–1.65 (m, 2H), 1.38–1.17 (m, 4H). EI-MS m/z (%): 247 .0(53) $[M]^+$, 204.0 (82), 167.0 (100), 128.0 (15), 81.1 (98), 41.1(18).

***In vitro* macrophage amastigote assay**

A recently developed promastigote rescue assay was used (Jain S, Sahu R, and Tekwani BL, unpublished). The THP1 cells (human acute monocytic leukemia cell line) were maintained in RPMI1640 medium supplemented with 10% fetal bovine serum (FBS). The cells were prepared prior to each assay and suspended in RPMI1640 medium with 10 % FBS at the cell density of 2.5 $\times10^5$ cells/mL. Phorbol 12-myristate 13-acetate (PMA) was added to the cell suspension to achieve a final concentration of 25 ng/mL, and cells were seeded onto a clear flat-bottom 96-well plate with 200 μL (5×10^4 cells) into each well. The plate was incubated in a 5% CO_2 incubator at

37 ℃ for at least 12 hours for differentiation of the THP1 cells to adherent macrophages. After overnight incubation, the medium from each well was discarded, and adherent cells were gently washed at least twice with serum free RPMI1640 medium. The *Leishmania donovani* promastigotes culture was harvested at the stationary phase (metacyclic infective stage) and suspended into RPMI1640 medium with 2% FBS at the density of 2.5×10^6 cells/mL. Then, 200 μL of promastigotes (5×10^5) culture was added to each well. The plate was further incubated in a 5% CO_2 incubator at 37℃ for at least 24 hours to allow infection of macrophages with the *Leishmania* parasites. After 24 hours, the non-adherent macrophages and unattached *Leishmania* promastigotes were washed off with serum free RPMI1640 medium. The infected macrophages were further incubated at 37℃ and 5% CO_2 in 200 μL RMPI1640 medium and 2% FBS with different concentrations of standard antileishmanial drugs (pentamidine & amphotericin B) or the test compounds for 48 hours. The control wells with medium, uninfected THP1 cells, infected cells without drugs, or test compounds were also set up simultaneously. The cultures were washed off with serum free RPMI1640 and treated for 30 seconds with 20 μL of 0.05% sodium dodecyl sulfate in RPMI1640 medium for the release of amastigotes from the infected macrophages. To each well, 180 μL of RPMI1640 medium with 10% FBS was added, and the plate was further incubated at 26℃ for 48 hours to allow transformation of released amastigotes to promastigotes. To each well, 20 μL of Alamar blue was added, and then the plates were incubated at 26 ℃ for 24 hours and read on a BMG Fluostar micropate reader (BMG Lab Technologies) at excitation wavelength of 544 nm and emission wavelength of 590 nm. Each compound was tested in duplicates at six concentrations; IC_{50} and IC_{90} values were computed from the dose-response curves.

Cytotoxicity assay

The *in vitro* cytotoxicity was determined against human monocytic leukemia cells (THP1) with a simple colorimetric method using the dye Alamar Blue, which was reported in the reference (Mikus, J.; Steverding, D. A simple colorimetric method to screen drug cytotoxicity against Leishmania, using the dye Alamar Blue. Parasitol Int. 2000, 48(3),265-269.). THP1 suspensions were grown in RPMI-1640 medium supplemented with 10% FBS, 2 mM glutamine, 50 μg/mL gentamicin and 0.0025 mg/L of amphotericin B (Sigma) at 37 °C in a 5% CO_2 atmosphere. Cells were grown to a density between 0.2 and 1×10^6 cells/mL. Culture medium was replaced every 2-3 days with fresh growth medium. DMSO was used as the solvent, and the test compounds was with six concentrations from 10-0.0032μg/mL.
